# Ameliorative Impacts of Wheat Germ Oil against Ethanol-Induced Hepatic and Renal Dysfunction in Rats: Involvement of Anti-Inflammatory, Anti-Apoptotic, and Antioxidant Signaling Pathways

**DOI:** 10.3390/life12101671

**Published:** 2022-10-21

**Authors:** Salwa A. Elgendy, Samar H. Baloza, Lina Abdelhady Mohammed, Hend Elsayed Nasr, Noha Osama El-Shaer, Heba I. Ghamry, Saed A. Althobaiti, Mustafa Shukry, Mohamed Mohamed Soliman, Heba A. Elnoury

**Affiliations:** 1 Department of Pharmacology, Faculty of Medicine, Benha University, Benha 13511, Egypt; 2 Genetic and Genetic Engineering, Animal Wealth Development Department, Faculty of Veterinary Medicine, Benha University, Benha 13736, Egypt; 3 Department of Medical Biochemistry and Molecular Biology, Faculty of Medicine, Benha University, Benha 13511, Egypt; 4 Physiology Department, Faculty of Medicine, Benha University, Benha 13511, Egypt; 5 Department of Home Economics, College of Home Economics, King Khalid University, P.O. Box 960, Abha 61421, Saudi Arabia; 6 Biology Department, Turabah University College, Taif University, P.O. Box 11099, Taif 21944, Saudi Arabia; 7 Department of Physiology, Faculty of Veterinary Medicine, Kafrelsheikh University, Kafrelsheikh 33516, Egypt; 8 Clinical Laboratory Sciences Department, Turabah University College, Taif University, P.O. Box 11099, Taif 21944, Saudi Arabia

**Keywords:** wheat germ oil, ethanol, oxidative stress, inflammation, gene expression

## Abstract

Wheat germ oil (WGO) is a well-known product with anti-inflammatory and antioxidant properties. The current study aimed to investigate the impacts of WGO against ethanol-induced liver and kidney dysfunction at the serum, anti-inflammatory, antioxidants and anti-apoptotic signaling pathways. Rats received saline orally as a negative control or WGO in a dose of 1.5 mL/kg (1400 mg/kg body weight orally) for 15 days. The affected group received ethanol 50% *v*/*v* 10 mL/kg (5 g/kg) body weight orally once a day for consecutive 15 days to induce hepatorenal injuries in ethanolic non-treated group. The protective group received WGO daily 1 h before ethanol administration. Serum (1.5 mL) from blood was extracted and examined for the changes in biochemical assessments in serum alkaline phosphatase (ALP), alanine aminotransferase (ALT), bilirubin, serum γ-glutamyl transpeptidase (GGT), total protein, serum albumin, butyrylcholinesterase (BChE), total cholesterol (TC), total triglyceride (TG), urea, creatinine, uric acid, potassium (K^+^), Beta-2 microglobulin (β_2_M), malondialdehyde (MDA), catalase (CAT), reduced glutathione (GSH), superoxide dismutase (SOD) and aspartate aminotransferase (AST). Kidney and liver homogenate was used to measure MDA, GSH and catalase activities. Quantitative real time PCR (qRT-PCR) was used to express Nrf2 and HO-1 in liver, and NF-kB and kidney injury molecule (KIM-1) in kidneys, which are correlated with oxidative stress and inflammation. Capase-3 and Bcl2 genes were examined using immunohistochemical analysis in the kidney and liver. Ethanol administration induced significant alteration in examined liver and kidney markers (AST, ALT, GGT, ALP, total proteins, urea, creatinine and uric acid). Moreover, alcohol administration decreased antioxidant activities at serum and hepatorenal tissues (GSH, catalase and SOD), while MDA was increased as a tissue degradation marker. Inflammatory cytokines, together with genes of oxidative stress markers (Nrf2 and HO-1), were all affected. At cellular levels, apoptotic marker caspase-3 was upregulated, while antiapoptotic marker B-cell lymphoma 2 (Bcl2), was down regulated using immunohistochemical analysis. Of interest, pretreatment with WGO improved the side effects induced by ethanol on hepatic, renal biomarkers and reversed its impact on serum and tissue antioxidant parameters. Nrf2/HO-1 were upregulated, while NFk-B and KIM-1 were downregulated using real time PCR. Immune reactivities of caspase-3 and Bcl2 genes were restored in the protective group. In conclusion, WGO ameliorated ethanol-induced hepatic and renal dysfunction at the biochemical, molecular and cellular levels by regulating some mechanisms that controls oxidative stress, apoptosis, inflammation and anti-apoptotic pathways.

## 1. Introduction

Globally, the most common form of chronic liver disease is alcohol-related liver disease (ALD). ALD can advance from alcoholic fatty liver (AFL) to alcoholic steatohepatitis (ASH), which is marked by hepatic inflammation. Chronic ASH may ultimately result in fibrosis, cirrhosis, and in rare cases, hepatocellular cancer (HCC). Additionally, severe ASH (with or without cirrhosis) may result in alcoholic hepatitis, an acute clinical manifestation of ALD that is linked to liver failure and a high death rate [1].

The development of ALD is influenced by gene variations that predispose to harmful alcohol drinking, and by individual susceptibility to develop advanced fibrosis [2].

There are no specific treatments for liver cirrhosis, however, avoiding alcohol is essential to slow the disease’s progression. Cirrhosis can develop into decompensated cirrhosis and hepatocellular cancer over time. Only a small number of carefully chosen patients are candidates for liver transplantation, and complete abstinence is a need. Liver transplantation may be useful for patients with decompensated liver cirrhosis and may also be utilized as a curative strategy for HCC [2,3]. ALD is a serious and fatal disease that affect various organs in our body especially liver and kidney [4] and one of the main causes of morbidity after cancer and cardiovascular diseases [5]. According to the World Health Organization, alcohol users were accountable for approximately 3.3 million dead people worldwide, or 5.9 percent of all fatalities [6]. The exact molecular and cellular mechanisms of tissue injury associated with chronic alcoholic consumption are highly complex and multifactorial [7].

Experimental and epidemiological research has shown that the quantity and frequency of alcohol consumption accelerate liver damage. Ethanol has an impact on every organ in the body due to its capability to penetrate all tissues as it has fat and water-soluble characters. The liver and kidney are the mainly affected organs by alcohol consumption [8]. The onset of ALD associates with chronic kidney disease; however, the effect of ethanol consumption on kidney function remains widely uninvestigated [7]. Hepatic and renal damage occurred by ethanol is believed to be triggered by oxidative stress [8]. Alcohol exposure can impair the liver and kidney functions in animal models [9]. Some heavy drinkers develop alcoholic hepatitis which is associated with a great mortality rate [10]. Chronic alcoholism also affects kidney filtration, causing rapid deterioration of kidney function with higher rate of mortality in hospitalized cases with alcoholic hepatitis rather than the major alcoholic hepatitis itself [11]. The liver is a main site of ethanol metabolism, as it executes several important mechanisms playing vital roles in digestion, storage and detoxification [8]. Chronic alcoholism may result in pathological alterations in cellular function caused by alcohol itself or its metabolites [12].

Acetaldehyde is the main toxic byproduct of ethanol that damages the liver when alcohol is oxidized by alcohol dehydrogenase (ADH) [8]. According to earlier studies, the degenerative effects of alcohol on several organs have been linked to increased oxidative stress, apoptosis, inflammation, and mitochondrial dysfunction [13,14]. Total serum protein deficiency with hypoalbuminemia and changes in hepatic enzyme activities are a specific finding in ALD [15]. Butyryl cholinesterase (BChE) is one class of cholinesterase enzyme which is synthesized by the liver and is abundant in the serum [16]. The decrease in BChE is used as an indicator of liver disorders [17]. Beta-2 microglobulin (β_2_M) is a polypeptide molecule, present on the surface of nucleated human cells. β_2_M is produced at a persistent rate and is eliminated by kidneys under physiological conditions. Elevated Β2M serum level is observed in renal diseases [18].

Despite the unlimited progress in the field, the development of appropriate drugs for the management of alcoholism remains a goal for alcohol studies. Natural products rich in bioactive compounds that are broadly well-known to have pharmacological uses, some of them are accountable for the prevention of oxidative stress [19]. Antioxidants were reported to improve the effect of oxidative stress and inflammation in liver-related disorders [20]. The use of natural antioxidant products has been extended worldwide due to their effectiveness and safety. They are widely used to treat several hepatic insults [21]. Therefore, exploration of an effective hepatoprotective agents will be a useful tool for the treatment of hepatic diseases.

Wheat germ is a byproduct of the milling of the grain into flour. The germ is kept separate from the bran and starch during the milling process [22,23]. The wheat germ, which makes up about 2.5 percent of the total weight and is essential in the production of foods with high nutritional value, is the most important component [24]. Wheat germ oil (WGO) consists of many tocopherols and phenol compounds, which have anti-inflammatory and antioxidant actions [25]. Additionally, it includes vitamin E and unsaturated fatty acids such as linolenic and linoleic acids that reduce the release of reactive oxygen species (ROS) in tissues [26]. The impacts of WGO against some toxicants were examined [27,28] using usual serum liver and kidney parameters but against ethanol that can be consumed in some populations, is still a subject for discussion, especially at the genetic and molecular levels.

Therefore, this study aimed to outline the possible mechanisms that may be incorporated in ameliorating the side effects induced by alcohol consumption on the liver and kidney. Various biochemical, molecular and cellular signaling pathways that regulate oxidative stress, apoptosis, inflammation, and anti-apoptotic markers were confirmed.

## 2. Materials and Methods

### 2.1. Chemicals and Kits

Alanine aminotransferases (ALT), aspartate aminotransferase (AST), alkaline phosphatase (ALP), serum γ-glutamyl transpeptidase (GGT), Bilirubin, urea, creatinine and uric acid were from Laboratory Bio-diagnostics Co., Cairo, Egypt. Serum Potassium (K^+^) level was estimated using kits supplied by Spectrum Diagnostics (Egyptian Co. for Biotechnology, Oboor City Industrial Area, Cairo, Egypt), Beta-2 microglobulin (β_2_M) using ELISA from Orgentec Diagnostica, Germany. Malondialdhyde (MDA) was from Life Span Biosciences Company (LS.Bio), North America, USA. While, Catalase (CAT), reduced glutathione (GSH) and superoxide dismutase (SOD) were from Shanghai Enzyme-linked Biotechnology Limited Company, Shanghai, China. Ethanol at 100% (St. Louis, MO, USA) was from Algomguria Co, Cairo, Egypt. Egypt’s El Captain Company in Cairo provided Wheat Germ Oil (WGO) (Cap Pharm). Hematoxylin and eosin (H&E) staining solution was obtained by Sigma Aldrich (St. Louis, MO, USA). The Oligod Tprimers, SYBR Green PCR Master Mix, and Qiazol were all supplied by QIAGEN (Valencia, CA, USA). All chemicals used were of molecular grade.

### 2.2. Animals and Experimental Design

Twenty-four adult male Wister rats (8-week-old weighing 180–200 g) were used for the current study. Animals were housed by adjusting room temperature at the Laboratory research of Pharmacology Department at Benha University and were handled manually for seven days to become totally adapted. The ethical rules for laboratory animal research were followed during all animal-related procedures based on the approval offered by Benha (RC.7.9.2022 N 000176) and King Khalid Universities. Rats were divided into four equal groups. Group I (control group): rats were given saline and unrestricted permission to water and food to evaluate the normal basic parameters. Group II, wheat germ oil group (WGO group): rats received WGO (1.5 mL/kg) (∼approximately 1.400 mg/kg) orally, every day [29] for 15 days. Group III (ethanolic group): rats were medicated with only ethanol 50% *v*/*v* (10 mL/kg) orally (about 5 g/kg), every day [30] for 15 days. Group IV (WGO + ethanol): rats were medicated with WGO (1.5 mL/kg) orally, every day [29] followed by ethanol 50% *v*/*v* (10 mL/kg) orally, 1 h later, every day for 15 sequential days [30]. After being anesthetized for 2 to 5 min in a desiccator with a cotton pad soaked in diethyl ether, the rats were scarified and killed by cervical dislocation. Blood samples were taken by a heart puncture, then centrifuged for 10 min at 3000 rpm to separate the serum. Serum was maintained at −20 °C for biochemical measurements while liver and kidney tissue specimens were taken for homogenization and oxidative stress measurements. Hepatic and renal tissue specimens were preserved with Qiazol for RNA analysis and real-time PCR. For tissue antioxidant measurements, an ice-cooled phosphate buffer was used, and Bowman’s solution was used for immunohistochemistry and histology.

### 2.3. Ethanol’s Administration and Preparation

In this study, the chronic dose of a 50 *v*/*v* ethanol solution was 5 g per kg body weight. Distilled water was used to dissolve 50 g of pure ethanol to make 100 mL. Each rat that was given ethanol treatment received the solution’s daily administration at a dose of 10 mL/kg (5 g/kg) for two weeks [30].

### 2.4. Hepatic and Renal Function Parameters Assay

The serum concentrations of AST, ALP, ALT, and GGT as well as urea, uric acid and creatinine were measured using kits imported from Bio-diagnostic Company, Dokki, Giza, Egypt. These serum biochemicals were measured using BIO-RAD spectrophotometer following the instructions provided with each kit [31,32], in line with the manufacturer’s instructions. GGT [33] and bilirubin [34]. Total serum proteins were measured by method of Lowry et al. [35], serum albumin by the method of Doumas et al. [36], serum potassium (K^+^) using atomic absorption spectrophotometer according to previous published paper [37] and serum β2M was measured using an ELISA kit.

### 2.5. Assessment of Serum Oxidant-Antioxidants and Cytokines

According to the instructions provided by the manufacturer, enzyme-linked immunosorbent assay (ELISA) kits for measuring MDA, catalase (CAT) and reduced glutathione (GSH) concentrations were acquired from Life Span Biosciences Company (LS.Bio), North America for MDA and Shanghai Blue Gene Biotech CO., LTD for CAT and GSH. CAT, GSH and MDA were measured based on methods described by [38,39,40], respectively. Interleukin-1 (IL-1) and tumor necrosis factor alpha (TNFα) serum levels were determined utilizing particular ELISA kits (ab255730 and ab46070, consecutively) and spectrophotometric analysis in accordance with the protocols provided by the kits. Using a commercial kit purchased from Abcam, USA, IL-10 was measured (Rat IL-10 ELISA Kit, ab100765). Data from the ELISA reader was estimated and analyzed in accordance with the directions supplied with the kit.

### 2.6. Assessment of MDA, GSH, and SOD Levels in Liver and Kidney Homogenate, with Metabolic Hepatic BChE, TC and TG Parameters

To obtain the supernatant from the homogenate, tissue was precisely measured (0.1 g), homogenized (10% homogenate), and centrifuged (4000 rpm, 4 °C, 10 min) [41]. BchE was determined in hepatic tissue by the method of Knedel and Bottger [42]. The corresponding kits were used to quantify total hepatic cholesterol (TC), triglyceride (TG) and MDA (Nanjing Jiancheng Bioengineering Institute, Nanjing, Jiangsu, China). The corresponding kits were used to measure CAT, SOD, and GSH in the liver and kidneys (Shanghai Enzyme-linked Biotechnology Limited Company, Shanghai, China) and were measured calorimetrically based on the information supplied with each kit.

### 2.7. Quantitative Real Time PCR (qRT-PCR) and Gene Expression in Hepatic and Renal Tissues

RNA was isolated by Qiazol reagents from the kidney and liver and was converted to cDNA using kits from Applied Bio systems, Waltham, Massachusetts, USA., which was run using SYBR Green master mix (Thermo scientific, Waltham, MA, USA). Table 1 demonstrates the primers’ list used for gene amplification. Data were validated using the ^2−ΔΔ^Ct formula [43] in the 7500 Fast system Real time PCR (Applied Bio systems, Waltham, Massachusetts, USA). Gene expression and intensity changes were determined by comparative cycle threshold (CT) values, normalized to β-actin.

### 2.8. Liver and Kidney Immunohistochemistry and Histology

Hepatic and renal specimens were cut into slices, dehydrated, and embedded in paraffin for histological analysis. Slices were then cut into 3 μm thick to be stained with hematoxylin and eosin (H&E), then visualized under an optical microscope.

Slices were embedded in paraffin, deparaffinized, and remoistened for immunohistochemical analysis. They were then cleaned in PBS and immersed for 15 min in 2 percent H2O2 to impede peroxidase activity. Bovine serum albumin (5%) was used to block non-specific binding sites. Bcl-2 Antibody [(C-2): sc-7382], Caspase-3 Antibody [(9CSP01): sc-81,663], and polyclonal antibodies (Santa Cruz Biotechnology, USA) were employed to coat the kidney and liver tissue specimen slides before being diluted to 1:500 and added. The slides were then kept at 4 °C for overnight incubation. A biotin-conjugated secondary antibody (catalog # sc-2040) was implemented to the slides after three PBS rinses. These were created with 3,3-diaminobezidine tetrahydrochloride, and hematoxylin was used as a counterstain [44]. The relative proportions of immune reactive cells for caspase-3 and Bcl2 measured by the ratio of positively stained cells to the total number of examined cells. Three slides from six rats in each group were the subjects of ANOVA tests to determine their significance.

### 2.9. Data Analysis

The data was tabulated and evaluated using statistical package for social sciences (SPSS) program was used to analyze the results (version 16, IBM Analytics, New York, NY, USA). The data were expressed as means and standard deviations (SD) for all investigated SEMs and biomarkers for all investigated genes. Employing the Shapiro-Wilk test with a normality level of *p* < 0.05, the data were examined for normality. To find variations between normally distributed data, the one-way analysis of variance (ANOVA) test was utilized. A significant ANOVA test was accompanied by post-hoc multiple comparisons utilizing Bonferroni testing to identify significant pairings. *p* < 0.05 was deemed significant in this study, and that was the accepted level of significance.

## 3. Results

### 3.1. Ameliorative Impacts of WGO on Liver, Kidney Biomarkers and on Hepatic BChE

There were liver and kidney dysfunction and damage in ethanolic groups as indicated by the significant elevation of serum hepatorenal biomarkers as AST, ALT, ALP, GGT, bilirubin, hepatic TC and TG, urea, creatinine, uric acid, K^+^ and Β2M with a decrease in total proteins, albumin and hepatic BChE (Table 2 and Table 3). Rats pretreated with WGO then ethanol showed improvement and there was a restoration of all altered parameters, as shown in Table 2 and Table 3.

### 3.2. Impacts of WGO on Serum Oxidant-Antioxidants Markers

**Figure 1:**
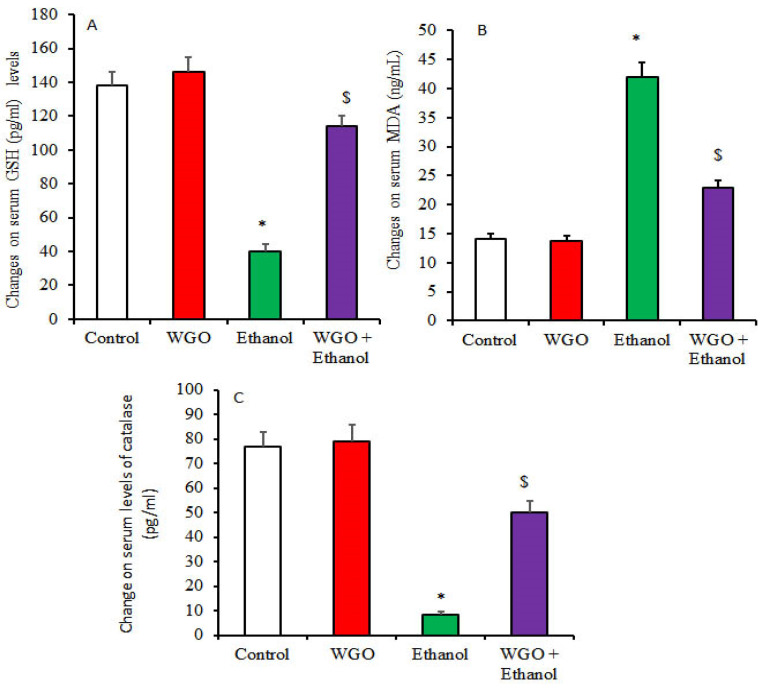
Protective effect of WGO on ethanol-induced systematic oxidative stress and changes in serum antioxidants [GSH (**A**), MDA (**B**), and catalase (**C**)]. Values are means ±SD for 6 different rats per treatment. Values are statistically different at *p* < 0.05. Symbol * means values are significant relative to control and WGO, and Symbol $ means values are significant relative to ethanolic group.

Figure 1 demonstrates an elevation in MDA levels, confirming tissue degradation and reduced serum catalase (CAT) and GSH levels in ethanolic non-treated rats, while rats which received WGO plus ethanol showed reduction in MDA with elevation in CAT and GSH levels, demonstrating that WGO had adapting ameliorative impact.

### 3.3. Impacts of WGO on Serum Inflammatory and Anti-Inflammatory Cytokines

**Figure 2:**
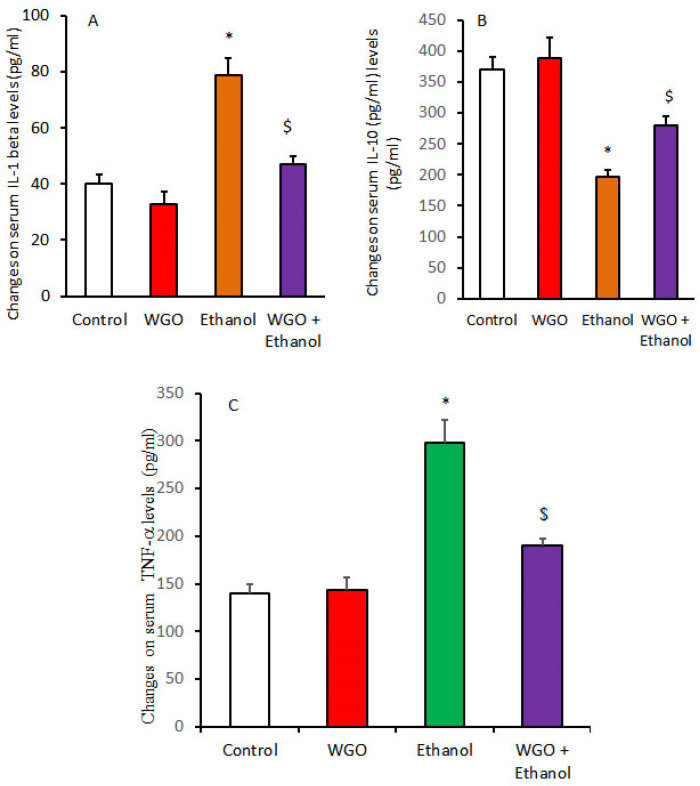
Protective effect of WGO on ethanol-induced changes in serum pro-inflammatory and anti-inflammatory cytokines levels [IL-1 beta (**A**), IL-10 (**B**), and TNF-alpha (**C**)]. Values are means ±SD for 6 different rats per treatment. Values are statistically different at *p* < 0.05. Symbol * means relative to control and WGO, and Symbol $ means relative to ethanolic group.

Ethanol administration resulted in significant increase in serum levels of inflammatory cytokines (TNF-α & IL-1β) with significant reduction in anti-inflammatory cytokine IL-10 compared to the control and WGO groups. WGO pre-administrated rats showed restoration of these parameters, as seen in Figure 2.

### 3.4. Impact of WGO on Kidney and Liver Homogenates’ Stress Markers

Table 4 shows that there was an increase in MDA levels with a decrease in CAT, GSH, and SOD levels in hepatic and renal homogenates from the ethanolic group compared to the normal and WGO rats. Pre-administration of WGO then ethanol showed significant improvement in altered oxidative stress biomarkers.

### 3.5. Effects of WGO on Quantitative Expression of (Nrf-2 and HO-1) in Liver Tissue

**Figure 3:**
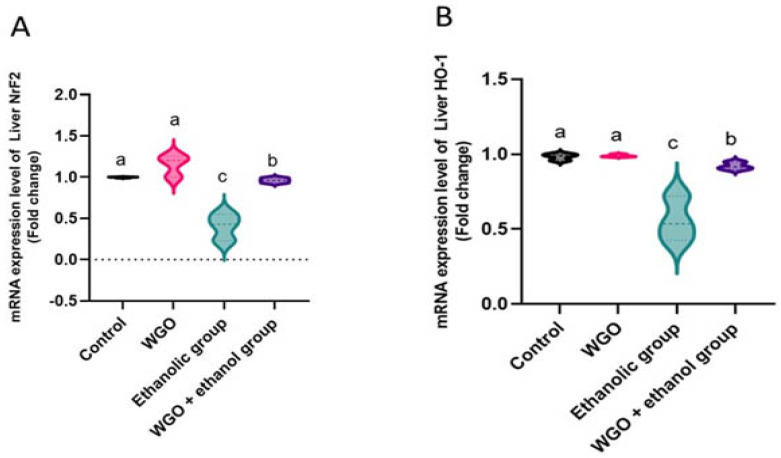
Quantification of Nrf2 (**A**) and HO-1 (**B**) mRNA expression in rat hepatic tissue of different groups after normalization with beta actin. Values reported as means ± SEM (*n* = 6). ANOVA test was followed by post hoc multiple comparisons test. Values with different letters are significant at *p* < 0.05.

HO-1 and Nrf-2 were down-expressed in liver tissue of ethanolic group compared with control and WGO rats (Figure 3A,B). The antioxidant efficiency appeared restored significantly in the protected group (WGO plus ethanol), as seen in Figure 3.

### 3.6. Effects of WGO on Quantitative Expression of NF-κB and KIM-1 in Renal Tissues

**Figure 4:**
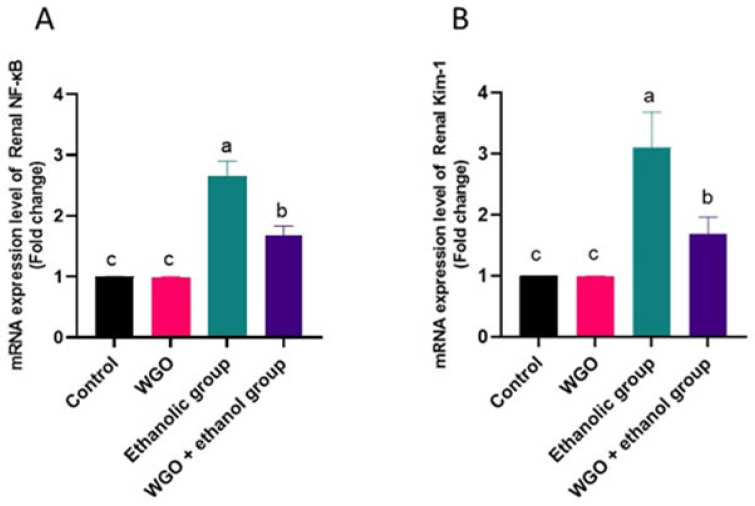
Quantification of NF_k_B (**A**) and KIM-1 (**B**) mRNA expression in rat kidney tissue of different groups after normalization with beta actin. Values are expressed as means ± SEM (*n* = 6). ANOVA test was followed by post hoc multiple comparisons test. At *p* < 0.05, values with different letters are significant.

NF-κB and KIM-1 mRNA expression were upregulated in renal tissues of ethanolic group (Figure 4A,B) compared to the control and WGO rats. In the protective group, renal NF-κB and KIM-1 expression was downregulated and restored in the WGO pre-administered rats.

### 3.7. Histopathological Examination

**Figure 5:**
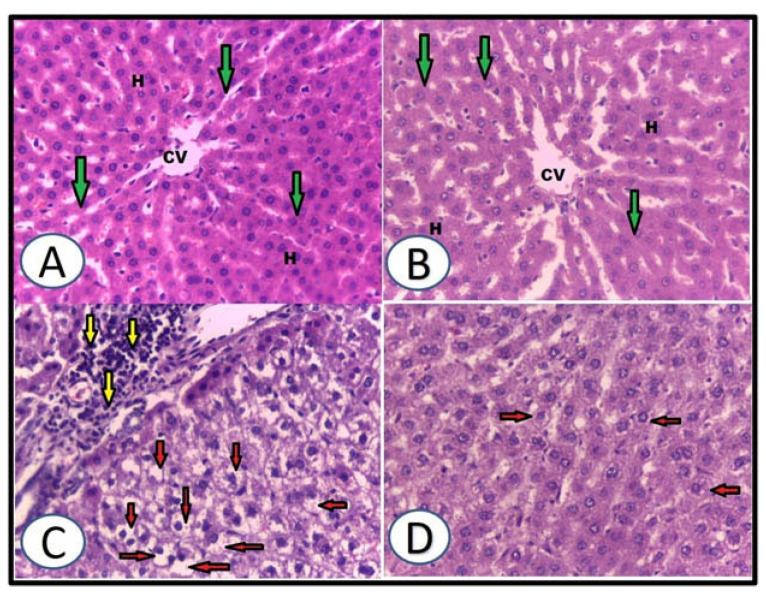
Impacts of WGO on liver histopathological changes induced by ethanol. (**A**): Liver of control group showed normal hepatocytes (H) with normal central vein (CV), hepatic cords (green arrow). (**B**): Liver of WGO administered group showed same histological picture. (**C**): Liver of ethanolic group showed diffuse hepatic steatosis (hepatocytes ballooning) (red arrow), inflammatory cells infiltration (yellow arrow) and interlobular deranged hepatic cord. (**D**): Liver of rats protected with WGO + ethanol showed mild degeneration of hepatocytes (red arrow) (H&E X400). Scale bar = 50 μm.

**Figure 6:**
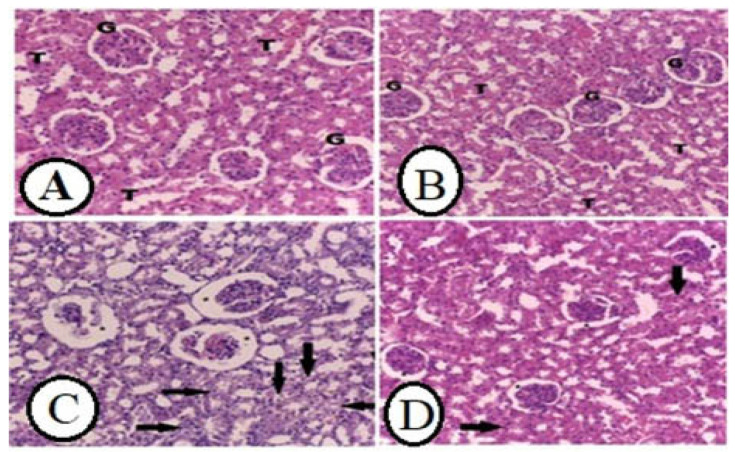
Impacts of WGO on renal histopathological changes induced by ethanol. (**A**): Normal control, (**B**): WGO treated rats showed normal glomeruli (G) and normal tubules (T). (**C**): Ethanolic rats kidney showed increase Bowman’s capsule space with glomerular shrinkage (*) and severe hydropic degenerative in the renal tubules (black arrow). (**D**): WGO + ethanol treated rat kidney showed mild hydropic degeneration of tubules (black arrow) with normal glomeruli (*) (H&E X200) Scale bar = 50 μm.

The experimental groups’ hepatic and renal histology was investigated in Figure 5 and Figure 6. The central vein, hepatic lobules, sinusoids, cords and the portal triad were demonstrated normally in the control rats (Figure 5A). The WGO-only rats displayed the same histology as well as control rats (Figure 5B), while ethanolic rats showed diffuse hepatic steatosis (hepatocytes ballooning), inflammatory cells infiltration and interlobular deranged hepatic cords (Figure 5C). In the protective group, WGO pretreatment plus ethanol rats showed mild degeneration of hepatocytes, indicating marked improvement of liver histological architecture (Figure 5D).

The control group’s kidney displayed normal renal structure, such as glomeruli and normal renal tubules (Figure 6A). The WGO only group displayed normal renal histological structure (Figure 6B), while the rats given ethanol demonstrated a dilated Bowman’s capsule, shrinking glomeruli, and obvious tubular hydropic degeneration (Figure 6C). On the other hand, WGO pretreatment plus ethanol showed marked improvement in renal histological structure (Figure 6D).

### 3.8. Caspase-3 and Bcl2 Immunohistochemistry

**Figure 7:**
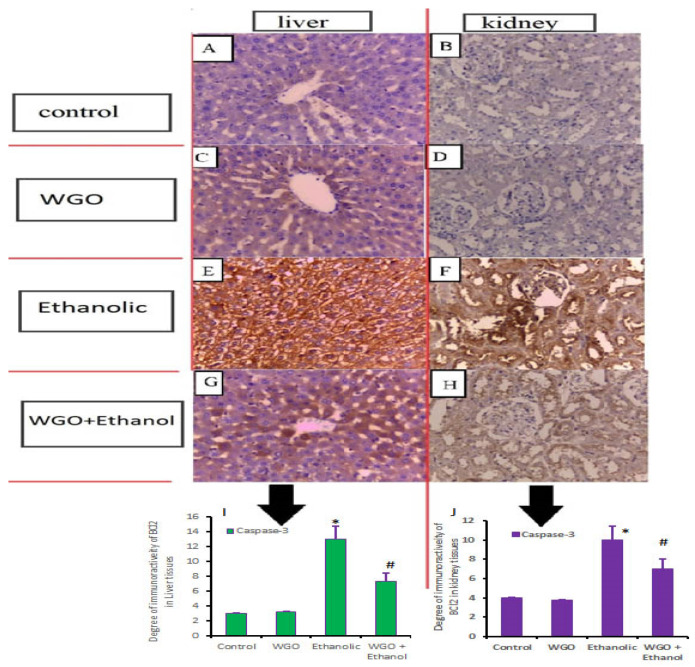
Impacts of WGO on the expression of caspase-3 proteins in liver and kidney after ethanol administration. Liver and kidney of control and WGO administered groups showed normal expression of caspase-3 (**A**–**D**) while ethanolic group showed high expression (**E**,**F**). Liver and kidney of rats protected with WGO then administered ethanol showed significant decline in caspase-3 expression (**G**,**H**) in comparison to ethanolic group. Immune positive reactivity is brown color. Scale bar = 50 μm. Protein expression were measured in immune histochemical analysis of liver and kidney tissue for caspase-3. Statistics was carried out one-way ANOVA followed by Tukey’s multiple comparisons test to examine the intensity of positive immune reactivity for caspase-3 in liver and kidney. In (**I**,**J**), the intensity of positive immune reactivity for caspase-3 in liver and kidney. The area percent (%) of caspase-3 immunohistochemical staining in 10 separate fields/sections was calculated, *n* = 6 rat/group. All values are expressed as means +SD and areas of expression with different letters are significant at *p* < 0.05. Symbol * means relative to control and WGO, and Symbol # means relative to ethanolic group.

**Figure 8:**
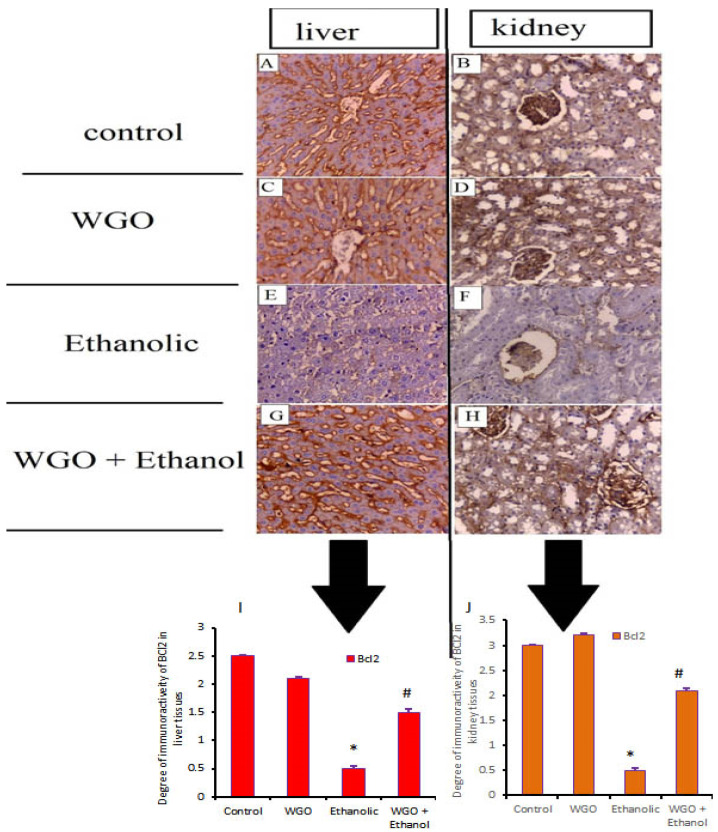
Impacts of WGO on hepatic and renal expression of Bcl2 proteins after ethanol administration. Liver and kidney of control (**A**,**B**) and WGO-administered groups (**C**,**D**) showed overexpression of Bcl2 in the hepatic parenchyma and renal tubules while ethanolic rats (**E**,**F**) showed significant downregulation in Bcl2 expression. Liver and kidney of rats protected with WGO then administered ethanol (**G**,**H**) showed significant restoration of Bcl2 expression in comparison to ethanolic group. Immune positive is brown color. Scale bar = 50 μm. Statistics was carried out one-way ANOVA followed by Tukey’s multiple comparisons test. In (**I**,**J**), the intensity of positive immune reactivity for Bcl2 in liver and kidney. The area percent (%) of Bcl-2 immunohistochemical staining in 10 separate fields/sections was calculated, *n* = 6 rat/group. All values are expressed as means +SD and areas of expression with different letters are significant at *p* < 0.05. Symbol * means relative to control and WGO, and Symbol # means relative to ethanolic group.

Figure 7A–H demonstrate alterations in caspase-3 expression in hepatic and renal tissues. The control rats showed normal expression for caspase-3 (Figure 7A,B), the same as in the WGO receiving rats (Figure 7C,D). The ethanolic group showed upregulation in caspase-3 expression (Figure 7E,F). On the other hand, WGO pretreatment showed significant reduction in immunoreactivity and expression of caspase-3 (Figure 7G,H). Therefore, it is very likely that WGO inhibits the caspase-3 upregulation occurred by ethanol. The collective densitometric immunoreactivity of caspase-3 in liver and kidney are shown in Figure 7I,J. Bcl2 expression and changes in hepatic and renal specimens are shown in Figure 8A–H. Bcl2 was abundantly expressed in the hepatic and renal parenchyma of control and WGO rats (Figure 8A–D) while there was downregulation of Bcl2 expression in ethanolic group (Figure 8E,F). Contrarily, Bcl2 expression was mostly restored in the WGO plus ethanol group (Figure 8G,H). The collective densitometric immunoreactivity of Bcl2 in the liver and kidney are shown in Figure 8I,J.

## 4. Discussion

The current study confirmed the ameliorative effects of WGO against ethanol-induced liver and kidney dysfunction. There was a significant increase in serum levels of AST, ALT, ALP, GGT, bilirubin, total hepatic cholesterol (TC), total hepatic triglyceride (TG), urea, creatinine, uric acid, serum K^+^ and Beta-2 microglobulin (β_2_M) with significant decrease in total protein, serum albumin and hepatic BChE. All were ameliorated when WGO was pre-treated to ethanolic rats. Moreover, there were genetic, histopathological and immunohistochemical changes in examined the liver and kidney of the ethanol receiving rats [8,45,46,47]. There was a close relation between excessive inflammatory cytokine generation, oxidative stress and alcohol-mediated hepatotoxicity and nephrotoxicity [48,49,50,51,52,53]. A previous report [27] is in agreement in some parts with ours as they examined the ameliorative impact of WGO on female rats with young age compared to rats used in current study. Here, we showed an increase in oxidative stress, lipid peroxidation with low antioxidant activity in the ethanol receiving group. The protective effect of WGO against various environmental toxicants were confirmed in rats which were administered sodium nitrite [27] and para-nonylphenol [28]. Both examined the reproductive hormones that were affected by sodium nitrite and para-nonylphenol, but in our study we gave our attention to liver and kidney biomarkers at enzyme levels, activities, hepatorenal oxidative stress markers, genes and cellular levels that were not examined by others [27,28]. The current study showed significant decrease in GSH, CAT, and SOD and also the lipid peroxidation’s induction (MDA) in the same group. Additionally, the expression of the genes for HO-1 and Nrf2 was down expressed in liver tissue of ethanolic rats. Our findings and recent studies [8,45,54,55] have shown that ethanol causes liver and kidney dysfunction through lipid peroxidation and redox dysfunction. On the other hand, pre-administration of WGO was found to increase antioxidant parameters in the tissues and serum of the liver and kidney while lowering oxidative stress biomarkers. Additionally, the expression of the genes HO-1 and Nrf2 was increased in the liver tissues, indicating a crucial function for WGO in the control of hepatic oxidative stress. Previous works [51,52,56] are on same line with ours in some parts reported pretreatment with WGO upregulated both HO-1 and Nrf2 expression rather than organs used (Stomach).

Oxidative damage induced by ethanol is closely related with elevated ROS generation [45] and reducing the activities of GSH and SOD [57]. They are the initial defense system’s line in the liver against oxidative damage and are free radicals scavengers for ROS [55]. The antioxidant response is regulated by Nrf2, which also regulates heme oxygenase-1 (HO-1; HMOX1) and cellular defense mechanisms [58]. This response comprises multiple downstream genes that are critical for regulating oxidative stress [59]. Nrf2 has been noted to act a basic role in defense versus oxidative stress [60] and that excessive ROS generation induces mitochondrial impairment, cytochrome c (cyt c) secretion, caspase stimulation and liver apoptosis [55]. Along with oxidative stress, steatohepatitis commonly occurrs with alcoholic consumption. Alcohol triggers the generation of pro-inflammatory cytokines, which worsens the inflammatory response and causes liver damage [61] and renal damage [47]. NF-κB is a member of transcriptional factors’ family that regulates the genes expression correlated with inflammation in retort to a variety of stimuli and is associated with multiple inflammatory responses [62]. Moreover, it controls a an array of pro-inflammatory cytokines involved in hepatocyte injury due to chronic alcohol consumption [63]. ROS-derived oxidative stress elevates the NF-κB expression [64].

Kidney injury molecule-1 (KIM-1) is a type I transmembrane glycoprotein that has been recognized as a biomarker of acute and chronic kidney disease [65]. During the repair of damaged renal tissue, (KIM-1) is greatly expressed at the proximal tubular epithelial cells’ apical membranes [66] associated with proximal tubular damage in clinical and experimental studies. Furthermore, in this research, ethanol administration resulted in significant rise in inflammatory cytokines’ serum level (TNF-α&IL-1β) [45,46] upregulation of NF-κB expression [47] and KIM-1 expression in renal tissues, also produced significant reduction in anti-inflammatory cytokine IL-10 [46,67], consequently led to increased inflammatory process. However, WGO pre-administration resulted in significant decrease in these inflammatory cytokines and NF-κB & KIM-1 expression in renal tissue with significant increase in anti -inflammatory cytokine. Similarly, these data were supported with previously published studies [53,56,68].

Furthermore, alcohol-induced liver impairment is marked by apoptotic signaling pathway [69] alcohol exposure causes cell death signaling cascades and apoptosis and impairments of organs function [70]. In our study, ethanol treatment upregulated caspase-3 (apoptotic genes) expression immune reactivity in both the liver and kidney, resulting in elevated apoptosis. On the contrary, Bcl-2 (anti-apoptotic genes) were inhibited by ethanol [14,47,71]. Alcohol stimulated the release of cytochrome c and reduced mitochondrial viability [72]. Previous studies have demonstrated that mitochondria play a vital role in regulation of alcohol-induced cell toxicity, inducing ROS production and inflammation [14,73]. Nevertheless, the pre-administration of WGO renovated the altered parameters to counteract, apoptosis induced by ethanol. Previous studies documented WGO has anti- apoptotic effect [50,52,56,74], proving that WGO has the potential to ameliorate gene expression associated with apoptosis in ethanol-induced liver and kidney damage in rats.

## 5. Conclusions

**Figure 9:**
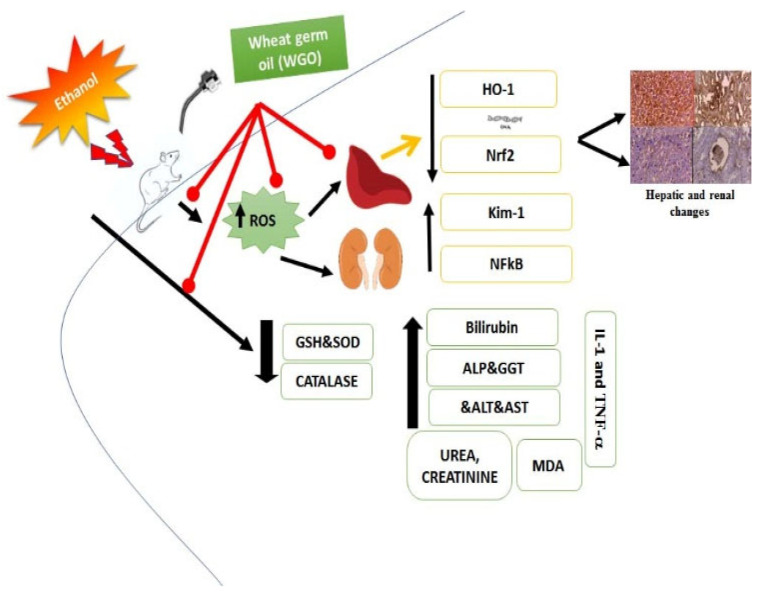
The collective preventive effects of WGO against ethanol-induced hepatic and renal damage in rats.

Current findings confirmed the mitigating impact of WGO on liver and kidney dysfunction induced by ethanol. Ethanol altered liver and kidney biomarkers, increased inflammatory cytokines, decreased antioxidants levels and altered different gene expression at liver and kidney level. The pre-administration of WGO retrieved all altered markers at biochemical, liver and kidney level. WGO regulated the expression of genes linked with antioxidants, inflammation and restored the genes that control caspase-3 and Bcl2 cellular immune reactivity. These results supported the potential use of WGO to protect liver and kidney against side effects of alcohol and open the field for further studies that search more signaling pathways may be involved is such regulations in other organs such as testis, brain and heart rather than kidney and liver. The collective effects of WGO against ethanol-induced induced hepatic and renal damage are shown in Figure 9.

## Figures and Tables

**Table 1:** Primers Oligonucleotide Sequences for q-PCR in liver and kidney of rat.

	Primers	5′—Oligo Sequences—3′	Accession Number
Liver	qR-Nrf2	F: TTGTAGATGACCATGAGTC R: TGTCCTGCTGTATGCTGCT	NM_031789.2
	qR-HO-1	F: GTAAATGCAGTGTTGGCCC R: ATGTGCCAGGCATCTCCTTC	NM_012580.2
Kidney	qR-KIM-1	F: TGGCACTGTGACATCCTCAGA R: GCAACGGACATGCCAACATA	NM_173149
	qR- NFkB	F: TCTCAGCTGCGACCCCG R: TGGGCTGCTCAATGATCTCC	AF079314
	qR-β actin	F: AAGTGTGACGTTGACATCCG R: TCTGCATCCTGTCAGCAATG	NM 031144

The sequences of rat primers; qR-Nrf2: nuclear factor erythroid 2-related factor 2 (Nrf2), qR-HO-1: hemoxygenase-1, qR-KIM-1: Kidney injury molecule-1, qR-NFkB: nuclear factor-kappa B (NF-kB) and β-actin was selected as the reference gene.

**Table 2:** Ameliorative impacts of WGO against Ethanol-induced changes liver and kidney biomarkers.

		Normal Control Group	WGO Group	Ethanolic Group	WGO + Ethanol Group
	ALT(IU/L)	26.86 ^a^ ± 5.55	27.73 ^a^ ± 6.9	64.63 ^b^ ± 7.86	38.37 ^c^ ± 2.78
	AST(IU/L)	73.073 ^a^ ± 3.67	75.43 ^a^ ± 4.25	187.33 ^b^ ± 10.01	105.70 ^c^ ± 8.52
Liver	ALP(IU/L)	80.09 ^a^ ± 3.73	77.43 ^a^ ± 4.25	165.44 ^b^ ± 4.22	114.83 ^c^ ± 7.73
	GGT(IU/L)	1.91 ^a^ ± 0.31	2.36 ^a^ ± 0.33	5.23 ^b^ ± 0.95	3.39 ^c^ ± 0.75
	Total bilirubin (mg/dL)	0.54 ^a^ ± 0.07	0.57 ^a^ ± 0.07	1.80 ^b^ ± 0.21	0.81 ^c^ ± 0.10
Kidney	Serum Urea (mg/dL)	33.51 ^a^ ± 2.60	32.18 ^a^ ± 3.45	58.63 ^b^ ± 7.83	43.10 ^c^ ± 3.72
	Serum Creatinine (mg/dL)	0.35 ^a^ ± 0.07	0.39 ^a^ ± 0.07	0.86 ^b^ ± 0.13	0.66 ^c^ ± 0.09
	Serum Uric acid (mg/dL)	1.08 ^a^ ± 0.42	1.27 ^a^ ± 0.51	5.01 ^b^ ± 1.38	2.71 ^c^ ± 0.53

Values are means ± SD for 6 different rats per treatment; Values with different letters are statistically different at *p* < 0.05; SD, standard deviation.

**Table 3:** Ameliorative impacts of WGO against Ethanol-induced changes in metabolic biomarkers of liver and kidney function.

		Normal Control Group	WGO Group	Ethanolic Group	WGO + Ethanol Group
Liver	Total protein (g/dL)	5.88 ^a^ ± 0.32	5.95 ^a^ ± 0.31	2.58 ^b^ ± 0.21	4.5 ^c^ ± 0.35
	Serum albumin (g/dL)	3.52 ^a^ ± 0.17	3.63 ^a^ ± 0.21	1.26 ^b^ ± 0.06	2.47 ^c^ ± 0.22
	Hepatic BChE (U/g wet tissue)	36 ^a^ ± 1.45	36.03 ^a^ ± 1.63	17.23 ^b^ ± 1.12	27.07 ^c^ ± 1.16
	TC (mmol/g protein)	3.22 ^a^ ± 0.31	3.05 ^a^ ± 0.11	7.38 ^b^ ± 1.02	5.22 ^c^ ± 0.95
	TG (mmol/g protein)	0.64 ^a^ ± 0.07	0.65 ^a^ ± 0.07	3.03 ^b^ ± 0.30	1.17 ^c^ ± 0.29
Kidney	Serum K^+^ (mEq/L)	3.53 ^a^ ± 0.21	3.63 ^a^ ± 1.03	6.05 ^b^ ± 0.34	5.0 ^c^ ± 0.35
	Serum β2 M (µg/dL)	1.15 ^a^ ± 0.09	1.21 ^a^ ± 0.05	6.5 ^b^ ± 0.35	4.08 ^c^ ± 0.32

Values are means ±SD for 6 different rats per treatment; Values with different letters are statistically different at *p* < 0.05; SD, standard deviation.

**Table 4:** Ameliorative impacts of WGO against Ethanol-induced oxidative stress in liver and kidney tissues.

		Normal Control Group	WGO Group	Ethanolic Group	WGO + Ethanol Group
	MDA (nmol/g protein)	2.24 ^a^ ± 0.75	2.22 ^a^ ± 0.74	6.41 ^b^ ± 1.53	4.06 ^c^ ± 0.36
Liver	CAT (U/g protein)	406.67 ^a^ ± 13.2	408.17 ^a^ ± 14.58	209.33 ^b^ ± 13.96	307.83 ^c^ ± 15.56
	GSH (U/g protein)	27.23 ^a^ ± 5.17	26.86 ^a^ ± 6.94	11.35 ^b^ ± 1.23	19.32 ^c^ ± 1.51
	SOD (U/g protein)	726.50 ^a^ ± 21.4	723.83 ^a^ ± 17.11	448.67 ^b^ ± 16.56	600 ^c^ ± 23.20
	MDA (nmol/g protein)	2.53 ^a^ ± 0.73	2.49 ^a^ ± 0.71	6.10 ^b^ ± 1.02	4.14 ^c^ ± 0.60
Kidney	CAT (U/g protein)	385.33 ^a^ ± 12.9	382.33 ^a^ ± 12.91	194.67 ^b^ ± 6.05	284.00 ^c^ ± 16.62
	GSH (U/g protein)	18.24 ^a^ ± 2.71	17.79 ^a^ ± 2.78	6.39 ^b^ ± 1.73	13.27 ^c^ ± 2.58
	SOD (U/g protein)	700.83 ^a^ ± 31.1	710.83 ^a^ ± 31.12	412.33 ^b^ ± 11.75	587.17 ^c^ ± 15.58

Values are means ±SD for 6 different rats per treatment; Values with different letters are statistically different at *p* < 0.05; SD, standard deviation.

## Data Availability

Data can be demanded upon request.
